# Etioepidemiological and Laboratory Profile of Tropical Fever in Patients Presenting With Acute Febrile Illness in Wardha District in Central India: An Observational Study

**DOI:** 10.7759/cureus.77861

**Published:** 2025-01-23

**Authors:** Sachin Agrawal, Maimoona Khan, Khadija F Hamdulay, Sunil Kumar, Avinash Parepalli, Rajvardhan Patil, Manikanta Nelakuditi

**Affiliations:** 1 Department of Medicine, Datta Meghe Institute of Higher Education & Research, Wardha, IND; 2 Internal Medicine, Jawaharlal Nehru Medical College, Wardha, IND

**Keywords:** acute febrile illness, dengue, leptospirosis, malaria, scrub typhus

## Abstract

Introduction

This study examined acute febrile illness (AFI) patients for several epidemiological and etiological factors. Acute febrile fever can result from various illnesses, including dengue, malaria, leptospirosis, and scrub typhus. The ranges of clinical outcomes of these cases were investigated as well.

Methods

This retrospective observational study was conducted from January 2023 to January 2024 for one year in the Medicine Department of Jawaharlal Nehru Medical College and Acharya Vinoba Bhave Rural Hospital, Datta Meghe Institute of Higher Education & Research (Deemed to be University), a tertiary care teaching medical college in Wardha District of Maharashtra in Central India. Six hundred patients with acute febrile illness who tested positive for leptospirosis, dengue, scrub typhus, and malaria were included in the study. A pre-made proforma was used to record the data.

Results

Out of 600 patients, 150 (25%) were dengue, malaria (62 (10.3%)), scrub typhus (45 (7.5%)) cases, and leptospirosis (41 (6.8%)) cases. In 150 cases of dengue, 63 (42%) had hepatitis as a complication at the time of presentation. Twenty-seven (43.55%) had presented with shock in 62 patients of malaria, 13 (31.71%) had acute respiratory distress syndrome (ARDS) and pneumonia in 41 cases of leptospira, and 13 (28.8%) had hepatitis in 45 cases of scrub typhus patients. Among 38 deaths, three (2%) died due to dengue, two (3.23%) due to malaria, one (2.44%) due to leptospirosis, one (2.44%) due to scrub typhus, and 31 (10.36%) due to acute febrile illness.

Conclusion

Dengue, malaria, scrub typhus, and leptospirosis were the four primary illnesses that led to AFI in hospitalized patients, with dengue being the most prevalent. Using a mix of clinical, epidemiological, and laboratory information, a pertinent action plan should be created to treat and prevent such fevers in any hospital setting.

## Introduction

The primary cause of febrile sickness in Southeast Asia's rural areas is tropical acute febrile illnesses (TAFIs), which also partially affect metropolitan areas. In these nations, there is ample evidence of TAFIs, which comprise chikungunya, leptospirosis, dengue fever, scrub typhus, Japanese encephalitis (JE), and malaria [1-2]. All age groups are susceptible to infectious diseases, but those who reside in tropical areas are especially affected. They are frequently spread by insect bites and brought on by various bacteria, viruses, and protozoa [3-4].

The assessment of the worldwide burden of neglected tropical illnesses, such as malaria, was 9.42 deaths per 100,000 people and 815.2 disability-adjusted to life-years per 100,000 people, according to statistics from the Institute for Health Metrics and Evaluation [5-6]. Compared to other vector-borne illnesses like malaria, which have seen significant drops in rates of 18 (%) worldwide, dengue has also contributed to a higher number of deaths worldwide, with 1.27 million recorded [7]. Over one million severe cases and 58,900 deaths in a year contribute to other tropical illnesses that are underreported, like leptospirosis [8-9].

People from the west of India (Maharashtra) have been reported to have several common tropical fevers, such as leptospirosis, scrub typhus, dengue, and malaria [2]. In our setting, treating and managing patients with febrile diseases with awareness and understanding of the main fevers and how numerous tropical fevers that present as AFIs coexist is critical. Season and geographic location have a major impact on the occurrence and trends of these tropical fevers in hospitalized patients [3-5]. Some of them are perennial, while others exhibit seasonal variation. Due to the similar clinical presentations of many tropical fevers, effective disease management demands expertise in the etiology and local epidemiology of these infections. Data regarding the incidence and etiology of these illnesses are scarce. There is currently relatively little information available on tropical fevers. In low-resource settings, fever may be treated empirically or self-treated since diagnostic tests are not readily available [2]. Understanding the local incidence of infections is crucial for directing clinical workup and treatment. The primary goal of this study was to characterize the causes of fever in patients at a central Indian tertiary care hospital. Secondary objectives included reporting on case fatalities and investigating correlations with clinical and demographic characteristics based on aetiological diagnosis.

## Materials and methods

This retrospective study was conducted in the Medicine Department of Jawaharlal Nehru Medical College and Acharya Vinoba Bhave Rural Hospital, Datta Meghe Institute of Higher Education & Research (Deemed to be University), Wardha, Maharashtra, India, after obtaining ethical clearance from the Institutional Ethics Committee (Ref. No. DMIHER (DU)/IEC/2024/374).

**Figure 1:**
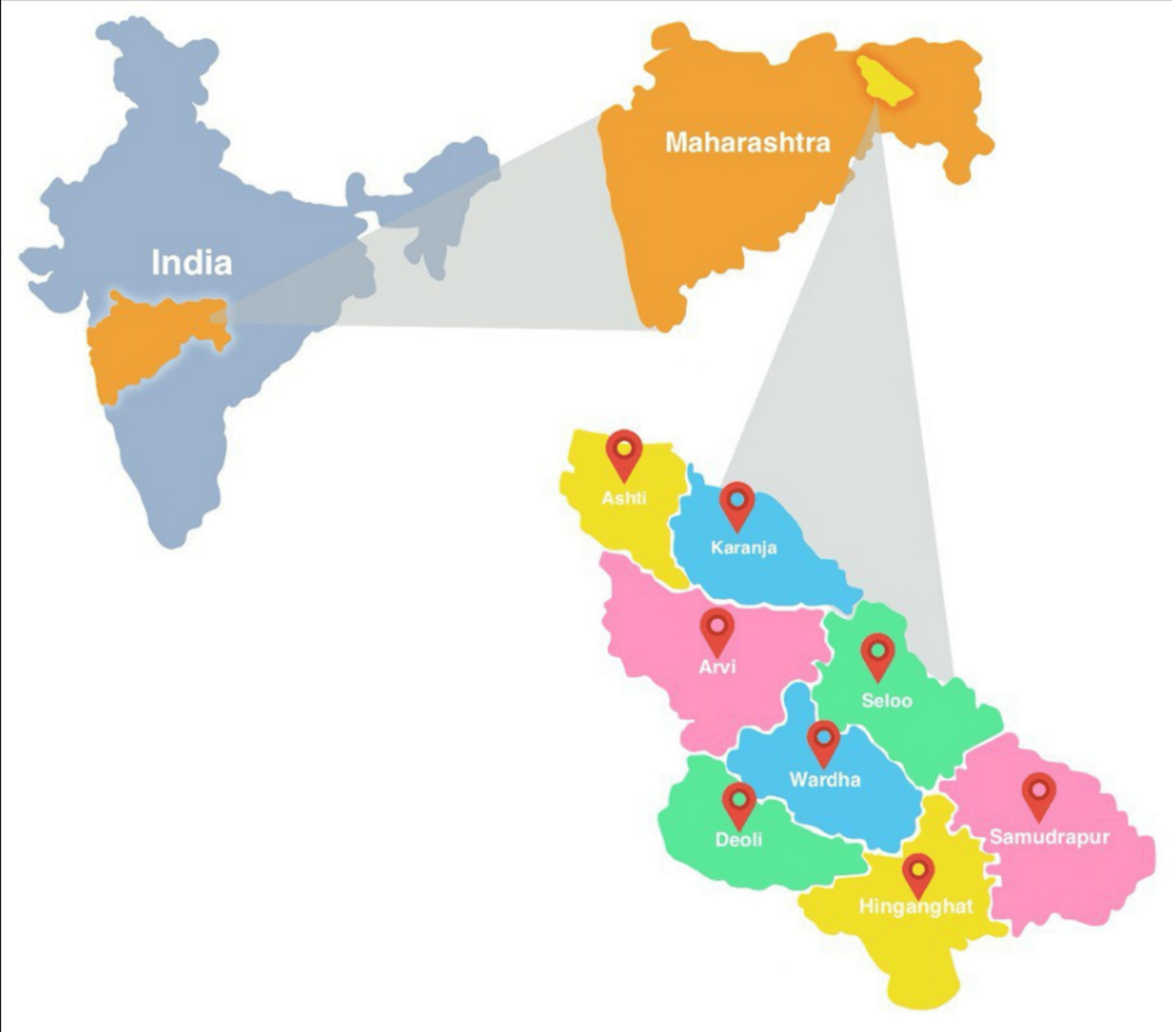
Schematic representation of collections of study sample from various regions of Wardha district from state of Maharashtra, India This figure is an original creation of the authors (Canva).

A total of 600 samples from suspected cases of AFIs were tested for leptospirosis, dengue, scrub typhus, and malaria, collected from various regions of Wardha district, as shown in Figure 1.

Baseline patient data included blood tests such as complete blood counts, renal profiles, liver function tests, venous blood gases, blood smears for malaria parasites, and both anaerobic and aerobic blood cultures. Dengue serology and NS1 antigen tests were selectively performed based on strict criteria, including residence in a dengue-prone area, the presence of clinical and biochemical indicators of viral fever, and blood count evidence of thrombocytopenia and leukopenia. The study included patients aged 18 years or older with a recorded temperature of 38°C or higher. Exclusion criteria comprised individuals under 18 years, those who did not provide consent, pregnant women, immunocompromised patients, individuals with chronic or nosocomial infections, and those with fever of non-infectious origin. Blood samples were collected for laboratory analysis of infectious agents, and a standardized data collection sheet (see Appendix) was used to record epidemiological, clinical, and biochemical information.

Definitions

Scrub typhus is a serious infectious illness brought on by the rickettsial bacteria *Orientia tsutsugamushi*. Larval trombiculid mite bites are the means of transmission, and they can cause end-organ damage, perivascular inflammation, vascular leakage, and widespread vasculitis. This disease, which can include fever, headache, myalgia, and gastrointestinal problems, can affect people of all ages, including travelers to endemic areas. Although its occurrence varies by location, an eschar, which is a black ulcer with core necrosis, may form at the bite site.

Chikungunya is a virus-borne illness spread by mosquitoes such as *Aedes aegypti *and *Aedes albopictus*. Fever, excruciating joint pain, joint swelling, muscular soreness, headache, nausea, exhaustion, and rash are its hallmarks, and it usually appears four to eight days after the bite.

When a person has laboratory proof of parasitemia but no malaria symptoms or indicators, this is confirmed asymptomatic malaria. When laboratory testing confirms parasitemia, a person with confirmed symptomatic malaria is diagnosed with the following symptoms: fever, back pain, headache, chills, diarrhea, myalgia, vomiting, and common indicators of splenomegaly and/or anemia. The presence of vivax or *Plasmodium falciparum*, or mixed trophozoites on thin and thick blood film smears, is indicative of laboratory-positive malaria in any patient.

Any instance where at least two of the following symptoms are present together with fever, such as vomiting, aches, rash, positive tourniquet test, leukopenia, and laboratory confirmation, is considered a confirmed case of dengue fever. Ideally, paired serum samples should be collected 10-14 days apart. A patient is deemed to have laboratory-positive dengue if they have a suggestive history and one of the following symptoms: (1) an acute-phase or convalescent-phase material due to a single positive anti-dengue virus IgM or (2) a positive dengue virus antigen detection by the NS1 rapid test. A leptospirosis case that has been confirmed is described as an acute febrile illness that manifests clinically with a history of possible exposure to the environment or contaminated water, as well as any one of the laboratory tests, which include positive cultures, microscopic agglutination tests (MAT), or the positive results of two (2) distinct rapid diagnostic tests. Undifferentiated acute febrile illness (UAFI) is the acute onset of febrile illness following a thorough investigation without a conclusive diagnosis based on a negative blood culture report and serology test.

Statistical analysis

Data were coded and recorded in MS Excel (Microsoft Corporation, USA). Descriptive statistics were used to summarize the clinical and sociodemographic characteristics of TAFI patients. Continuous variables were presented as means and standard deviations, while categorical data were reported as frequencies and percentages. Median/means were used to elaborate the descriptive statistics and IQRs/SDs for continuous and categorical variables; percentages/frequencies were used. Differences in the group for categorical data were compared with the help of the chi-square test/Fisher's exact test. Statistical analysis was carried out using IBM SPSS Statistics for Windows, Version 23.0 (released 2015, IBM Corp., Armonk, NY). Statistical significance was set at p < 0.05.

## Results

A total of 600 patients who met the selection criteria were included in the study. Out of all of these, the following were identified as the etiologic agents of tropical fever: dengue (150 cases), malaria (62 cases), scrub typhus (45 cases), and leptospirosis (40 cases). There were 347 men and 253 women, as shown in Table 1.

**Table 1:** Distribution of study subjects according to age and gender (n = 600) AFI: acute febrile illness. Not significant, p > 0.05

Age group (years)	Dengue n = 150	Malaria n = 62	Leptospira n = 41	Scrub n = 45	AFI n = 302	Total 600 (100%)	ϰ2-value	p-value
18-40 years	96 (64%)	37 (59.68%)	26 (63.41%)	22 (48.89%)	192 (63.58%)	373 (62.17%)	8.97	0.34 = not significant
40-60 years	41 (27.33%)	16 (25.81%)	13 (31.71%)	14 (31.11%)	81 (26.82%)	165 (27.50%)		
>60 years	13 (8.67%)	9 (14.52%)	2 (4.88%)	9 (20%)	29 (9.60%)	62 (10.33%)		
Gender								
Male	83 (55.33%)	35 (56.45%)	28 (68.29%)	27 (60%)	174 (57.62%)	347 (57.83%)	2.36	0.66 = not significant
Female	67 (44.67%)	27 (43.55%)	13 (31.71%)	18 (40%)	128 (42.38%)	253 (42.17%)		

In 150 cases of dengue, 63 (42%) had hepatitis as a complication at the time of presentation. Twenty seven (43.55%) presented with shock in 62 patients of malaria, 13 (31.71%) had ARDS and pneumonia in 41 cases of leptospira, and 13 (28.8%) had hepatitis in 45 cases of scrub typhus patients. Other complications are highlighted in Table 2.

**Table 2:** Distribution of study subjects according to complications (n = 600) AFI: acute febrile illness, AKI: acute kidney injury, ARDS: acute respiratory distress syndrome, DIC: disseminated intravascular coagulation Significant, p < 0.05

Complications	Dengue n = 150	Malaria n = 62	Leptospira n = 41	Scrub typhus n = 45	AFI n = 302	Total 600 (100%)	ϰ2-value	p-value	
AKI	34 (22.67%)	9 (14.52%)	7 (17.07%)	10 (22.22%)	3 (0.99%)	63 (10.50%)	905.44		0.0001 Significant
Shock	2 (1.33%)	0 (0%)	0 (0%)	0 (0%)	0 (0%)	2 (0.33%)			
ARDS	7 (4.67%)	11 (17.74%)	13 (31.71%)	0 (0%)	0 (0%)	31 (5.17%)			
DIC	27 (18%)	0 (0%)	0 (0%)	10 (22.22%)	0 (0%)	37 (6.17%)			
Encephalopathy	1 (0.67%)	0 (0%)	0 (0%)	0 (0%)	0 (0%)	1 (0.17%)			
Hepatitis	63 (42%)	9 (14.52%)	8 (19.51%)	13 (28.89%)	0 (0%)	93 15.50%)			
Pneumonia	15 (10%)	6 (9.68%)	13 (31.71%)	1 (2.22%)	0 (0%)	35 (5.83%)			
Shock	1 (0.67%)	27 (43.55%)	0 (0%)	11 (24.44%)	0 (0%)	39 (6.50%)			
Not any	0 (0%)	0 (0%)	0 (0%)	0 (0%)	299 (99.01%)	299 (49.83%)			

In 150 cases of dengue, 93 (62%) had fever, 97 (64.67%) had headache, 93 (62%) had pain in the abdomen, 86 had vomiting (57.33%), 93 (62%) had bleeding, 44 (29.33%) had pallor, 104 (69.33%) had jaundice, 121 (80.67%) had edema in the lower limb, 78 (52%) had pleural effusion, 62 (41.33%) had ascitis, 80 (53.33%) had hepatomegaly, 121 (80.76%) had splenomegaly, and 117 (78%) had rash at the time of presentation. All patients of AFIs had a fever. Clinical features in other tropical fevers are highlighted in Table 3.

**Table 3:** Distribution of the study subjects according to symptoms and signs (n = 600) Edema LL: edema of the lower limb, AFI: acute febrile illness Significant: p < 0.05, non-significant: P > 0.05

Symptoms	Dengue, n = 150		Malaria, n = 62		Leptospira, n = 41		Scrub, n = 45		AFI, n = 302		Total, 600 (100%)		ϰ2-value	p-value
	N	%	N	%	N	%	N	%	N	%	N	%		
Fever	93	62.00	22	35.48	41	100.00	36	80.00	111	36.75	303	50.50	92.20	0.0001 = significant
Headache	97	64.67	39	62.90	30	73.17	36	80.00	198	65.56	400	66.67	5.21	0.26 = non-significant
Pain in the abdomen	93	62.00	28	45.16	19	46.34	32	71.11	157	51.99	329	54.83	12.44	0.014 = significant
Vomiting	86	57.33	21	33.87	0	0.00	45	100.00	138	45.70	290	48.33	97.35	0.0001 = significant
Bleeding	93	62.00	62	100.00	4	9.76	45	100.00	118	39.07	322	53.67	154.22	0.0001 = significant
Pallor	44	29.33	15	24.19	23	56.10	38	84.44	196	64.90	316	52.67	89.47	0.0001 = significant
Jaundice	104	69.33	0	0.00	15	36.59	45	100.00	176	58.28	340	56.67	132.34	0.0001 = significant
Edema LL	121	80.67	0	0.00	7	17.07	26	57.78	199	65.89	353	58.83	153.88	0.0001 = significant
Eschar	60	40.00	10	16.13	0	0.00	45	100.00	163	53.97	278	46.33	119.76	0.0001 = significant
Pleural effusion	78	52.00	0	0.00	26	63.41	2	4.44	140	46.36	246	41.00	87.54	0.0001 = significant
Ascitis	62	41.33	29	46.77	41	100.00	9	20.00	121	40.07	262	43.67	65.30	0.0001 = significant
Hepatomegaly	80	53.33	30	48.39	19	46.34	0	0.00	134	44.37	263	43.83	41.28	0.0001 = significant
Splenomegaly	121	80.67	62	100.0	17	41.46	0	0.00	173	57.28	373	62.17	144.03	0.0001 = significant
Rash	117	78.00	25	40.32	41	100.00	0	0.00	0	0.00	183	30.50	408.18	0.0001 = significant

Of the 600 patients, 562 underwent successful treatment and were discharged. Of the 38 deaths recorded, three (2%) were attributed to dengue, two (3.23%) to malaria, one (2.44%) to leptospirosis, one (2.44%) to scrub typhus, and 31 (10.36%) to acute febrile illness, as shown in Table 4.

**Table 4:** Distribution of the study subjects according to outcome (n = 600) AFI: acute febrile illness Significant: P < 0.05, non-significant: P > 0.05

Outcome	Dengue n = 150	Malaria n = 62	Leptospira n = 41	Scrub n = 45	AFI n = 302	Total 600 (100%)	ϰ2-value	p-value
Death	3 (2%)	2 (3.23%)	1 (2.44%)	1 (2.22%)	31 (10.26%)	38 (6.33%)	15.95	0.0001 = significant
Discharged	147 (98%)	60 (96.77%)	40 (97.56%)	44 (97.78%)	271 (89.74%)	562 (93.67%)		

All dengue patients were NS1- and IgM-positive; all malaria cases were para-check positive, and all leptospirosis and scrub typhus cases were IgM positive, as shown in Table 5.

**Table 5:** Distribution of study subjects according to test results (n = 600) AFI: acute febrile illness Significant:p < 0.05, non-significant: p > 0.05

Test results	Dengue n = 150	Malaria n = 62	Leptospira n = 41	Scrub typhus n = 45	AFI n = 302	Total 600 (100%)	ϰ2-value	p-value
NS1	150 (100%)	0 (0%)	0 (0%)	0 (0%)	0 (0%)	150 (25%)	527.17	0.0001 = significant
IgM	150 (100%)	0 (0%)	41 (100%)	45 (100%)	0 (0%)	236 (39.33%)	600	0.0001 = significant
IgG	2 (1.33%)	0 (0%)	5 (12.20%)	5 (11.11%)	0 (0%)	12 (2%)	6.02	0.19 = non-significant
Overall	302 (50.50%)	0 (0%)	46 (7.67%)	50 (8.33%)	0 (0%)	398 (66.33%)		

Lab parameters of dengue, malaria, leptospira, scrub typhus, and AFI are seen in Table 6. Among the 150 dengue cases, a considerable proportion of the cases, 86 (57.33%), have platelet counts ranging from 50,000 to 1 lakh, indicating moderate severity in most individuals. Furthermore, 49 (32.67%) patients have platelet counts below 50,000, which is problematic since low platelet levels can lead to serious sequelae from dengue fever. Only 14 (9.33%) patients have platelet counts of 1 to 1.5 lakh, with only one (0.67%) over 1.5 lakh. This data demonstrates that the majority of dengue cases result in reduced platelet counts. The remaining parameters are seen in Table 6.

**Table 6:** Distribution of study subjects according to lab parameters (n = 600) Significant (S): p < 0.05, non-significant (NS): p > 0.05

Lab parameters	Dengue, n = 150		Malaria, n = 62		Leptospira, n = 41		Scrub, n = 45		AFI, n = 302		Total, 600 (100%)		ϰ2-value
	N	%	N	%	N	%	N	%	N	%	N	%	
Hb%													
8-10	11	7.33	8	12.90	2	4.88	4	8.89	30	9.93	55	9.17	8.68 P = 0.37, NS
10-12	33	22.00	17	27.42	7	17.07	14	31.11	86	28.48	157	26.17	
>12	106	70.67	37	59.68	32	78.05	27	60.00	186	61.59	388	64.67	
TLC													
<4000	27	18.00	10	16.13	3	7.32	3	6.67	73	24.17	116	19.33	16.64 P = 0.034, S
4000-11000	105	70.00	45	72.58	36	87.80	35	77.78	198	65.56	419	69.83	
>11000	18	12.00	7	11.29	2	4.88	7	15.56	31	10.26	65	10.83	
Platelet count													
<50000	49	32.67	0	0.00	3	7.32	0	0.00	7	2.32	11	1.83	21.65 P = 0.012, S
50000-1 lakh	86	57.33	1	1.61	0	0.00	3	6.67	20	6.62	38	6.33	
1-1.5 lakh	14	9.33	19	30.65	14	34.15	12	26.67	88	29.14	182	30.33	
>1.5 lakh	1	0.67	42	67.74	24	58.54	30	66.67	187	61.92	369	61.50	
Red blood cell (RBC)													
<140	89	59.33	38	61.29	41	100.00	45	100.00	302	100.00	515	85.83	181.38 P = 0.0001, S
140-200	61	40.67	24	38.71	0	0.00	0	0.00	0	0.00	85	14.17	
>200	-	-	-	-	-	-	-	-	-	-	-	-	
Blood urea													
<40	65	43.33	24	38.71	21	51.22	21	46.67	141	46.69	272	45.33	2.16 P = 0.70, NS
>40	85	56.67	38	61.29	20	48.78	24	53.33	161	53.31	328	54.67	
Creatinine													
<1.2	62	41.33	38	61.29	28	68.29	25	55.56	153	50.66	306	51.00	13.53 P = 0.009, S
>1.2	88	58.67	24	38.71	13	31.71	20	44.44	149	49.34	294	49.00	
Sodium													
<135	64	42.67	29	46.77	19	46.34	14	31.11	127	42.05	253	42.17	4.97 P = 0.76, NS
135-140	38	25.33	13	20.97	12	29.27	12	26.67	81	26.82	156	26.00	
>140	48	32.00	20	32.26	10	24.39	19	42.22	94	31.13	191	31.83	
Potassium													
<3.5	-	-	-	-	-	-	-	-	-	-	-	-	7.73 P = 0.10, NS
3.5-5	111	74.00	53	85.48	30	73.17	40	88.89	227	75.17	461	76.83	
>5	39	26.00	9	14.52	11	26.83	5	11.11	75	24.83	139	23.17	
Sr. bilirubin													
<1.2	29	19.33	34	54.84	15	36.59	20	44.44	98	32.45	196	32.67	29.11 P = 0.0001, S
>1.2	121	80.67	28	45.16	26	63.41	25	55.56	204	67.55	404	67.33	
Prothrombin time international normalized ratio (PT INR)													
<1.3	150	100	62	100	41	100	45	100	302	100	600	100	-
>1.3	-	-	-	-	-	-	-	-	-	-	-	-	-
Aspartate transaminase (AST)													
< 2 time	49	32.67	24	38.71	12	29.27	28	62.22	141	46.69	254	42.33	23.36 P = 0.002, S
2-4 times	21	14.00	11	17.74	7	17.07	8	17.78	45	14.90	92	15.33	
>4 times	80	53.33	27	43.55	22	53.66	9	20.00	116	38.41	254	42.33	
Alanine transaminase (ALT)													
< 2 time	63	42.00	23	37.10	8	19.51	23	51.11	114	37.75	231	38.50	17.74 P = 0.023, S
2-4 times	14	9.33	2	3.23	1	2.44	2	4.44	28	9.27	47	7.83	
>4 times	73	48.67	37	59.68	32	78.05	20	44.44	160	52.98	322	53.67	
C-reactive protein (CRP)													
<10	8	5.33	13	20.97	13	31.71	10	22.22	64	21.19	108	18.00	24.52 P = 0.0001, S
>10	142	94.67	49	79.03	28	68.29	35	77.78	238	78.81	492	82.00	

## Discussion

A significant issue in the world's tropical regions is tropical fever. A sizable portion of food-borne or vector-borne tropical illnesses are found in India. These tropical fevers are challenging to detect clinically because they frequently manifest as AFI with similar signs and symptoms. Our assessment identified scrub typhus, Dengue, and malaria as the most common causes of AFI among patients. These results align with similar studies conducted in South East Asian countries, including India [6-10].

The high number of dengue cases in our study could be attributed to the endemic nature of the disease in our region. One of the primary factors driving the spread and establishment of urban mosquitoes, including *Aedes aegypti*, is the urbanization of rural areas. Previous studies have highlighted leptospirosis and scrub typhus as leading causes of AFI in Southeast Asia [11-15]. Scrub typhus is responsible for roughly 10-19% of all acute undifferentiated fever cases studied in this region; leptospirosis is a less frequent cause [16-21]. The trend shift may be due to high point-of-care diagnostics identifying endemic diseases like malaria and dengue. Scrub typhus and leptospirosis, however, have been overlooked because most hospital laboratories lack the necessary diagnostic tests. Financial assistance is a significant element in screening for these neglected diseases, as is the lack of concern about feverish illness among administrators, laboratory staff, and physicians. In addition, only tertiary hospitals can access investigations like leptospirosis serology and IFA that do not use point-of-care testing.

The most prevalent clinical feature was fever, which was followed by rashes, myalgia or widely used body pain, and vomiting. In 62% of dengue cases and 80% of scrub typhus cases, fever was present. This suggests that some scrub typhus and dengue cases might not have been afebrile when first reported. Furthermore, 7.5% and 25% of the patients had scrub typhus and dengue virus infections, usually resulting in acute fever. Our study was conducted during the post monsoon. Scrub typhus is more prevalent during cooler months, and dengue outbreaks are reported in these months [22-24]. In 150 cases of dengue, 93 (62%) had fever, 97 (64.67%) had headache, 93 (62%) had pain in the abdomen, 86 (57.33%) had vomiting, 93 (62%) had bleeding, 44 (29.33%) had pallor, 104 (69.33%) had jaundice, 121 (80.67%) had edema in the lower limb, 78 (52%) had pleural effusion, 62 (41.33%) had ascitis, 80 (53.33%) had hepatomegaly, 121 (80.76%) had splenomegaly, and 117 (78%) had rash at the time of presentation than those reported by other workers in our region [11-13]. In malarial cases, the most significant were headache (39, 62.90%), splenomegaly (62, 100%), pain in the abdomen (28, 45%), and vomiting (21, 33%) at the time of presentation than those reported by other workers in our region [4-13].

In leptospirosis, 41 (100%) had fever, 30 (73.17%) had headache, 19 (46%) had pain in the abdomen, 15 (36%) had jaundice, 19 (46.34%) had hepatomegaly, and 26 (63%) had pleural effusion at the time of presentation than those reported by other workers in our region [5,8,9,17]. In the scrub, 36 (80%) had fever, headache, and eschar (45, 100%) at the time of presentation than those reported by other workers in our region. In AFI, 100% had fever, 198 (65.56%) had headache, 157 (51.99%) had pain in the abdomen, 199 (65.89%) had edema in the lower limb, and 173 (57.28%) had splenomegaly at the time of presentation than those reported by other workers in our region. Various authors had reported CNS involvement, manifesting as either meningitis or encephalitis in all these AFIs [25-27]. Neurological involvement as encephalopathy was found to be one in dengue (0.67%), three in malaria (5%), two in leptospira (4.87%), three in scrub typhus (6.66%), and 10 in AFIs (3.31%). While some studies have looked at different clinical and laboratory indicators to distinguish between tropical fevers, others have looked at establishing a scoring system that can distinguish between different kinds of undifferentiated fevers [8,16,28,29]. In tropical fevers, liver involvement may be elucidated by hepatocyte apoptosis that impaired liver caused directly by the infectious agent fluid leakage leading to perfusion, oxidative immune-mediated injury, or stress.

Of the 600 patients, 562 underwent successful treatment and were discharged. Of the 38 deaths recorded, three (2%) were attributed to dengue, two (3.23%) to malaria, one (2.44%) to leptospirosis, one (2.44%) to scrub typhus, and 31 (10.36%) to acute febrile illness. Hepatitis, shock, acute kidney injury (AKI), ARDS, pneumonia, encephalopathy, and disseminated intravascular coagulation (DIC) were the complications in our study. This finding could have been affected by the higher dengue prevalence in our study, as dengue is known to be a significantly more deadly illness among AFIs.

As per the Ministry of Health and Family Welfare, Government of India, malaria, dengue fever, and chikungunya caused substantial mortality in Maharashtra in 2020. Malaria deaths were 12 out of 12,909 verified cases. Dengue caused 10 fatalities among 3,356 confirmed cases. Chikungunya did not kill anyone, although it did result in 782 confirmed cases. Wardha District fared well, with no malaria or chikungunya-related deaths or morbidities reported. However, dengue fever caused 110 instances of sickness but no fatalities [30].

Limitations

There were some restrictions on this retrospective study. Since our hospital is a tertiary care facility, the reported complication rates may have a far higher referral bias. The single-centric nature of this study means that patient selection and referral patterns may be biased toward a particular group of patients. The lack of resources prevented gold routine tests for these different AFIs from being carried out for confirmation.

## Conclusions

Tropical fevers that impact patients in our area include dengue, malaria, scrub typhus, and leptospirosis. This retrospective study determines scrub typhus as a growing problem in our setup. This information will be extremely beneficial to clinicians as it will increase their awareness of the different symptoms associated with these tropical fevers. The outcomes of this research and subsequent studies on AFIs should be highly beneficial when developing a suitable plan of action to address this public health issue.

## Figures and Tables

**Figure 2:**
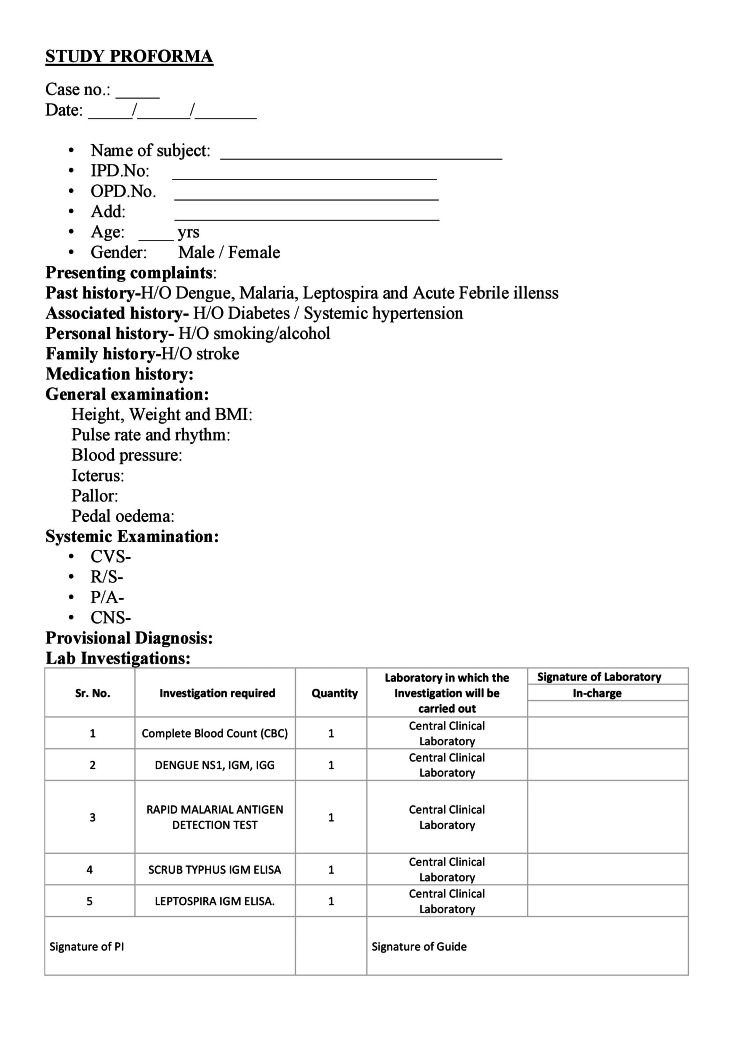
Pre-made proforma
